# Production of the medaka derived from vitrified whole testes by germ cell transplantation

**DOI:** 10.1038/srep43185

**Published:** 2017-03-03

**Authors:** Shinsuke Seki, Kazunari Kusano, Seungki Lee, Yoshiko Iwasaki, Masaru Yagisawa, Mariko Ishida, Tadashi Hiratsuka, Takao Sasado, Kiyoshi Naruse, Goro Yoshizaki

**Affiliations:** 1 Bioscience Education and Research Support Center, Akita University, 1-1-1 Hondo, Akita, Akita 010-8543, Japan; 2 Department of Marine Bioscience, Tokyo University of Marine Science and Technology, 4-5-7 Konan, Minato-ku, Tokyo 108-8477, Japan; 3 Laboratory of Bioresources, National Institute for Basic Biology, 38 Saigo-naka, Myodaiji-cho, Okazaki, Aichi 444-8585, Japan

## Abstract

The medaka (*Oryzias latipes*) is a teleost model distinguished from other model organisms by the presence of inbred strains, wild stocks, and related species. Cryopreservation guarantees preservation of these unique biological resources. However, because of their large size, cryopreservation techniques for their eggs and embryos have not been established. In the present study, we established a methodology to produce functional gametes from cryopreserved testicular cells (TCs). Whole testes taken from medaka were cryopreserved by vitrification. After thawing, the cells dissociated from cryopreserved testicular tissues were intraperitoneally transplanted into sterile triploid hatchlings. Some cells, presumably spermatogonial stem cells, migrated into the genital ridges of recipients and resulted in the production of eggs or sperm, based on sex of the recipient. Mating of recipients resulted in successful production of cryopreserved TC-derived offspring. We successfully produced individuals from the Kaga inbred line, an endangered wild population in Tokyo, and a sub-fertile mutant (*wnt4b*^−/−^) from cryopreserved their TCs. This methodology facilitates semi-permanent preservation of various medaka strains.

The medaka (*Oryzias latipes*) is native to East Asia. It has been used as an experimental animal for more than 100 years^1^. More than 10 unique inbred strains have been derived from genetically distinctive wild populations^2^. In addition, whole genome has been sequenced for five inbred strains^3^,^4^. Furthermore, gain-of-function by cytoplasmic DNA injection into zygotes^5^ and loss-of function by targeted mutagenesis with zinc finger nucleases (ZFNs)^6^, transcription activator-like effector nucleases (TALENs)^7^, and clustered regularly interspaced short palindromic repeats/CRISPR associated proteins (CRISPR/CAS9)^8^ enable us to establish numerous transgenic lines and mutants. Thus, the medaka is an excellent vertebrate model for a wide range of disciplines, including developmental biology, genetics, physiology, neurobiology, ecotoxicology, and evolutionary biology. Currently, 11 inbred strains, 67 wild populations, 22 related species belonging to genus *Oryzias*, 297 mutants, and 85 trangenics are available to the worldwide scientific community from the Japanese National BioResource Project^9^.

The wild populations of medaka have been declining because of habitat destruction, including river construction projects completed without consideration of their impacts on ecosystems^10^. In addition, the wild medaka is threatened by introduced exotic predators, such as largemouth bass (*Micropterus salmoides*), smallmouth bass (*M. dolomieu*), and bluegill (*Lepomis macrochirus*)^11^. Genetic contamination caused by mating with the introduced ornamental medaka is another threat^12^. Because of these threats, in 1999, the medaka was listed in the Red List of Threatened Animals of Japan^13^. Furthermore, 10 of 15 species belonging to the genus *Oryzias* (for example, *O. curvinotus* in China and Vietnam, *O. nigrimas* in Indonesia, *O. monogenesis* in Thailand, and *O. dancena* in India, among others) are also listed as endangered species according to the IUCN (International Union Conservation of Nature) Red List^14^. The IUCN is the global authority on the status of the natural world and the measures needed safeguard it. Thus, the conservation of these endangered species is vital to the prevention of extinction.

The only currently available method for preserving medaka strains is rearing live individuals and cryopreservation of their sperm^15^. Compared with mice and rats, the medaka is associated with higher risks of loss of parent fish through pathogen infection or accidental fatalities related to rearing facilities because of the water pumps and heaters in the breeding facilities. Moreover, there is an unavoidable problem of mutation caused by mobile DNA elements^16^,^17^. Indeed, genomic change caused by transposition of the *Albatross* transposon in the *Double anal fin* strain has been observed^18^.

In some mammals, including mice and rats, cryobanking of embryos and sperm^19^ can semi-permanently preserve genetic resources; however, past attempts in the cryopreservation of fish embryos and mature eggs have been unsuccessful^20^ because of their large size and low membrane permeability^21^,^22^.

Therefore, in the present study, we focused on spermatogonia that are sufficiently small (10-μm diameter) compared with 70–100-μm diameter mammalian embryos, which can be cryopreserved. Our group previously transplanted cryopreserved rainbow trout (*Oncorhynchus mykiss*) spermatogonia into recipient masu salmon (*O. masou*). The transplanted germ cells differentiated into fully functional eggs and sperm in the recipient salmon ovaries and testes, respectively. By mating the recipient males and females, we produced offspring ultimately derived from frozen cells^23^. However, this protocol has never been applied to experimental fish species. In the present study, we established a protocol for the cryopreservation of whole testis of the medaka, possessing spermatogonia, by vitrification, since vitrification can be performed quickly without expensive equipments, as long as liquid nitrogen is available. Next, we investigated whether the cryopreserved medaka spermatogonia could be differentiated into functional eggs and sperm in recipient gonads after spermatogonial transplantation. Finally, we applied this system in bioresource projects to produce offspring of an inbred medaka strain, endangered wild medaka, and mutant medaka with low fertility from vitrified testes.

## Results

### Optimization of cryopreservation conditions for whole testis of medaka by vitrification

**Figure 1:**
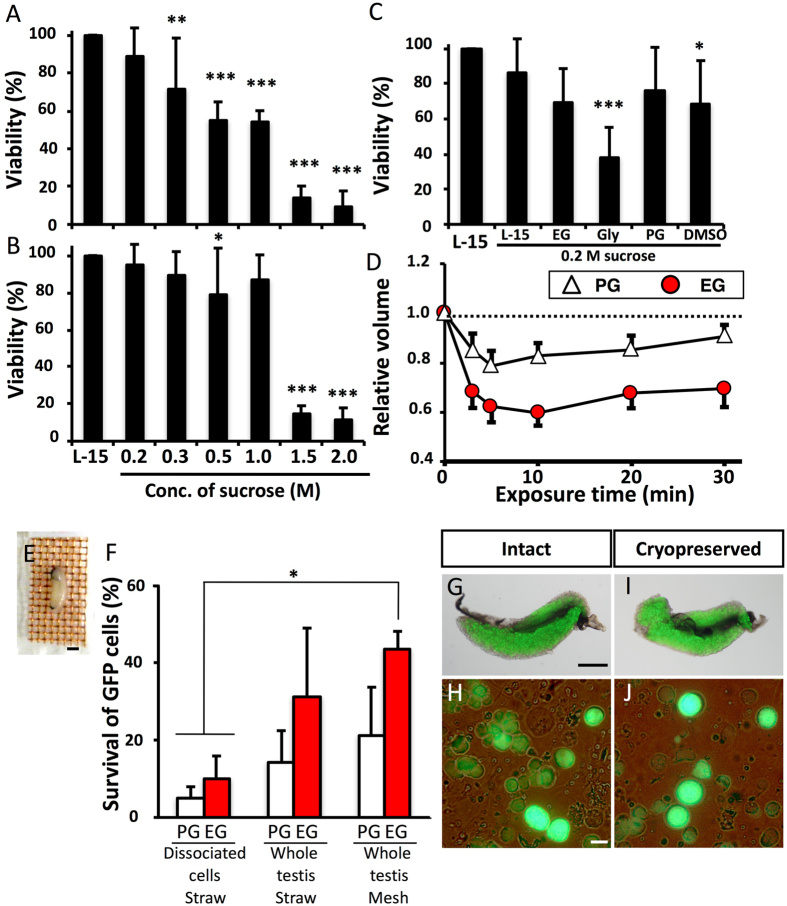
Optimization of cryopreservation conditions for whole testes of medaka by vitrification. (**A,B**) Viability of testicular cells after exposure to a hypertonic solution at 25 °C for 10 min (A) (n = 7) and at 0 °C for 30 min (**B**) (n = 9). Viability of testicular cells after exposure to each cryoprotectant solution at 25 °C for 30 min followed by a 0.2 M sucrose solution at 0 °C for 5 min (**C**) (n = 8). Volume changes in testicular cells following 30 min at 0 °C in each cryoprotectant solution (**D**) (n = 5–6). Testes on copper mesh (**E**). Survival of GFP-positive cells in testicular cells after cryopreservation with a vitrification solution (21% [w/v) ficoll and 0.35 M sucrose], including 30% (v/v) PG or 30% (v/v) EG in a plastic straw or on copper mesh (**F**) (n = 8). Testes and testicular cells (*olvas-GFP*) without cryopreservation (**G,H**) and after cryopreservation (**I,J**). Data are shown as the mean ± SEM (*P < 0.05; ***P < 0.001). Scale bars, 1 mm (**E,G**), and 10 μm (**H**).

To vitrify the cells and retain high viability, it is essential to prevent intracellular ice formation during cooling and warming by dehydration and permeation of a cryoprotectant. When testes were dehydrated using 0.2–1.0 M sucrose (0.5–1.8 Osm kg^−1^, Fig. 1A) at 25 °C for 10 min, the survival of green fluorescent protein (GFP)-positive germ cells was significantly lower than that of the non-treated control; however, the survival rates were improved when dehydration was carried out at 0 °C for 30 min (Fig. 1A,B). Toxicity assay for various cryoprotectants (−10%, 25 °C) revealed that propylene glycol (PG) was the less toxic, whereas ethylene glycol (EG) was also less toxic than glycerol and dimethyl sulfoxide (DMSO) (Fig. 1C). Dehydration (shrinkage) and permeation of cryoprotectant (to their previous volume) in PG and EG solutions was confirmed by measuring changes in germ cell volume in each solution (Fig. 1D). Based on these results, vitrification solutions containing 21% (w/v) ficoll and 0.35 M sucrose were based with either 30% (v/v) PG or 30% (v/v) EG and the testes were vitrified after exposure to them at 0 °C for 20 min. When dissociated cells were cryopreserved in plastic straws, survival was low, at approximately 10% in both EG and PG. When whole testes were cryopreserved in the straws, survival increased. In addition, when warming was conducted more rapidly with the use of a copper mesh, a survival rate of 44.4% was obtained using the vitrification solution containing EG, although the survival of cells treated with PG remained low (Fig. 1E). Medaka whole testes could be cryopreserved by direct immersion in liquid nitrogen after exposure to vitrification solution (including 30% (v/v) EG, 21% (w/v) ficoll, and 0.35 M sucrose) at 0 °C for 20 min with a copper mesh. Whole testes on a copper mesh were warmed by direct immersion in 0.2 M sucrose at 25 °C, followed by immersion in 0.2 M sucrose solution at 0 °C, and whole testes were immersed in L-15 medium. Thus, we successfully developed a simple cryopreservation method for spermatogonia in whole testis without the need of a complicated device, such as a programmable freezer.

### Production of oocytes derived from vitrified testicular cells (TCs) via transplantation into triploid female recipients

**Figure 2:**
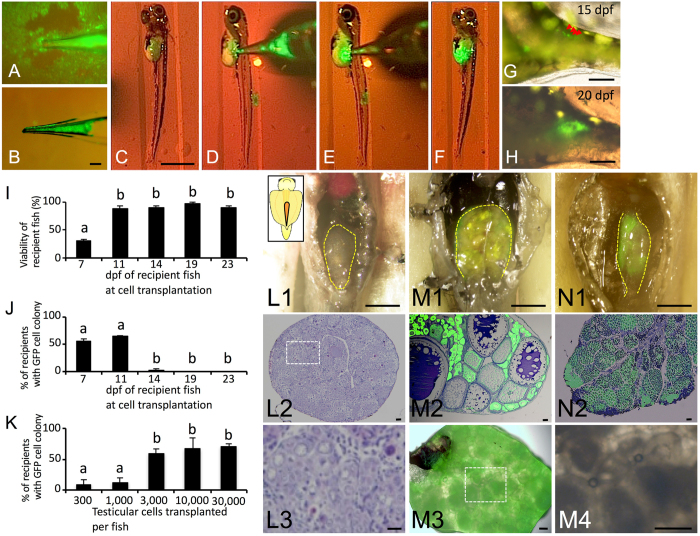
Transplantability of testicular cells retrieved from cryopreserved whole testes. (**A–F**) Intraperitoneal transplantation of donor testicular cells (*olvas-GFP*) into a recipient hatchling under a fluorescence microscope. (**G,H**) Donor-derived TCs (arrow) showing green fluorescence were incorporated into recipient gonads at 15 dpf (**G**) and incorporated TCs began to proliferate at 20 dpf (**H**). (**I**) Survival rates of recipients at each developmental stage after transplantation. (**J,K**) The incorporation rate of GFP-positive cells in recipient gonads for each developmental stage of the recipient (**J**) and those for each cell number (**K**). Data are shown as the mean ± SEM (Different letters indicate significant differences, P < 0.01). (L1-3, L3 is magnified photograph of L2) Gonads of WT female triploid. (**M,N**) Proliferation of donor germ cells in a triploid recipient ovary (M1-4, M4 is magnified photograph of M3) and testes (N1-2) at two months. Scale bars, 1 mm (**C**), 100 μm (B, G, H, L1, M1, M3-4, N1), and 10 μm (L2, L3, M2, N2).

Because technologies for the cryopreservation of fish eggs or embryos have not been available to date, the production of functional eggs derived from cryopreserved materials is important. Therefore, we determined whether transplanted TCs could be incorporated into ovaries of female recipients and differentiated into functional eggs. Whole testes of the *olvas-GFP* medaka on a copper mesh were vitrified by direct immersion in liquid nitrogen after exposure to vitrification medium including 30% (v/v) EG, 21% (w/v) ficoll, and 0.35 M sucrose at 0 °C for 20 min. After thawing the whole testes, the 15,000 dissociated TCs were transplanted into the body cavity of hatching fry at 11 days post fertilization (dpf) (Fig. 2A–F). At 15 dpf, transplanted TCs migrated to the recipient’s genital ridges (Fig. 2G) and propagated in the recipient gonads at 20 dpf (Fig. 2H).

To optimize the conditions for transplantation, proper timing of TC transplantation and optimum cell numbers were determined. The survival of recipients was approximately 90% when the transplantation was conducted at 11–23 dpf, although that of 7 dpf (3–4 days before hatching) was quite low (20%, Fig. 2I). The colonization efficiency was high when transplantation was performed on 7 dpf larvae and 11 dpf newly hatched larvae, but transplanted TCs were not incorporated into the recipient’s gonads when transplantation was performed after 14 dpf (Fig. 2J). Although colonization efficiency was approximately 10% with 300 or 1,000 cells transplanted into hatchlings at 11 dpf, it was 60% when >3,000 cells were transplanted (Fig. 2K) and efficiency was not further increased with the use of 10,000 or 30,000 cells.

When the TCs were transplanted into triploid recipients, which cannot produce their own eggs (Fig. 2L1, 2, 3), the fertility of the recipients was rescued and the triploid recipients produced only donor-derived oocytes with GFP fluorescence (Fig. 2M1, 2, 3). In male recipients, transplanted TCs could be incorporated into the gonads (Fig. 2N1, 2). Thus, we successfully induced the recipients to produce eggs derived from cryopreserved TCs.

### Production of donor-derived offspring from vitrified TCs

**Figure 3:**
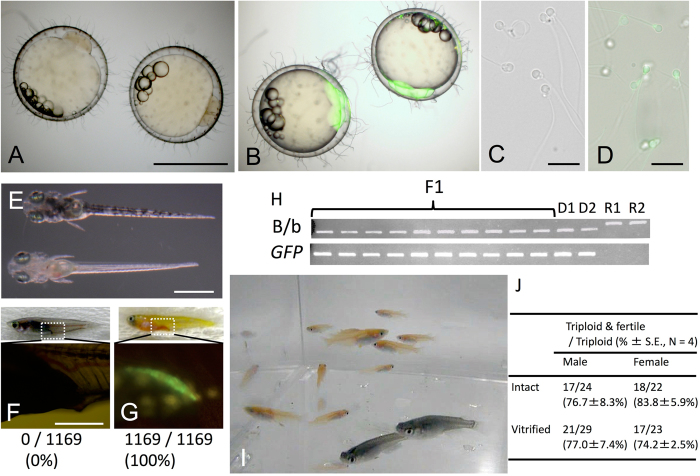
Production of functional gametes and medaka individuals derived from cryopreserved whole testes. (**A**) Embryos obtained from WT parents. (**B**) Embryos obtained from triploid recipients after transplantation of cryopreserved *olvas-GFP* TCs. (**C**) Sperm obtained from WT male medaka. (**D**) Sperm obtained from male recipients. (**E,F,G**) A hatching WT juvenile (top of **E** and **F**). A hatching juvenile derived from mating of recipients displaying the donor-derived phenotype of an orange body color (bottom of **E**) and gonads containing GFP-positive germ cells (**G**), suggesting that all F1 offspring were donor-derived. (**H**) PCR analysis, performed with *tyrosinase*-specific primers and *gfp*-specific primers, of F1 offspring derived from WT triploid recipients by cryopreserved *olvas-GFP* TC transplantation (F1), donor *olvas-GFP* medaka (D1-2) and WT recipient medaka (R1-2). (**I**) Efficiency of triploid and TC transplantation. Data are shown as the mean ± SEM. (**J**) one-month-old medaka (orange colored *olvas-GFP* medaka) generated from surrogate triploid WT parents by cryopreserved TC transplantation. Scale bars, 1 mm (**A,E**), 100 μm (**G**), and 10 μm (**C**).

We next determined whether the donor-derived eggs produced by the triploid recipient could be ovulated and fertilized. Based on mating studies, female triploid recipients that received cryopreserved TCs ovulated and produced fertilizable eggs. Their embryos had blastodiscs exhibiting clear green fluorescence (Fig. 3B). In males, triploid recipients successfully produced sperm showing green fluorescence, suggesting that the sperm was derived from cryopreserved donor TCs, which were presumably the spermatogonia (Fig. 3D). To determine whether the donor-derived haplotype was transmitted to the F1 generation, body color and green fluorescence of F1 juveniles were determined by progeny tests of recipient males and females. In this mating experiment, the donor was the *olvas-GFP* orange homozygous medaka and the recipient was the non-transgenic black medaka. Because the orange color phenotype is recessive to black color, if either sperm or oocytes were of recipient origin, the F1 medaka would exhibit the black phenotype. If both sperm and oocytes were derived from transplanted donor TCs, the orange medaka would be obtained. The matings produced larvae exhibiting the orange phenotype (Fig. 3E bottom). Further, to confirm their genetic origin, the gonad of the F1 orange medaka was observed under a fluorescent microscope. Green florescence, caused by *olvas-GFP*, was clearly detected in their germ cells (Fig. 3G). When 1,169 F1 medaka obtained by mating of the male and female recipients were observed, all exhibited the orange phenotype. The causal gene of the orange phenotype^24^ was also detected by PCR. Moreover, using PCR, we confirmed that 10 randomly selected F1 medaka carried the *GFP* gene derived from the donor (Fig. 3H). All F1 juveniles exhibited phenotype and genotype identical to those of the donor medaka (GFP-positive and orange-colored), which indicated that the F1 offspring obtained from triploid transplant recipients were all derived from donor-cryopreserved testes (Fig. 3I). When intact or cryopreserved 15,000 TCs were transplanted, 76.7 ± 8.3% or 83.8 ± 5.9% (N = 4) of triploid male recipients produced only sperm derived from donor and 77.0 ± 7.4% or 74.2 ± 2.5% (N = 4) of triploid female recipients produced only oocytes derived from donor, respectively (Fig. 3J; supplementary Table 1; supplementary Table 2). The remaining triploid recipients remained infertile.

To examine functionality of donor-derived eggs and sperm produced by triploid wild type recipients, the progeny test was conducted with three pairs of male and female triploid recipients that received *olvas-GFP* TCs. Fertilization rates of the resulting ovulated eggs (fertilization rate 91.6 ± 2.1% [N = 3], 91.6 ± 7.2 fertilized embryos/102.3 ± 8.0 ovulated eggs in a week) was not significantly different from those obtained from control WT males and females (fertilization rate 94.2 ± 1.1% (N = 3), 106.3 ± 17.7 fertilized embryos/112.7 ± 17.7 ovulated eggs).

### Production of offspring derived from vitrified TCs of inbred line (Kaga) and endangered wild populations (Tokyo-medaka)

**Figure 4:**
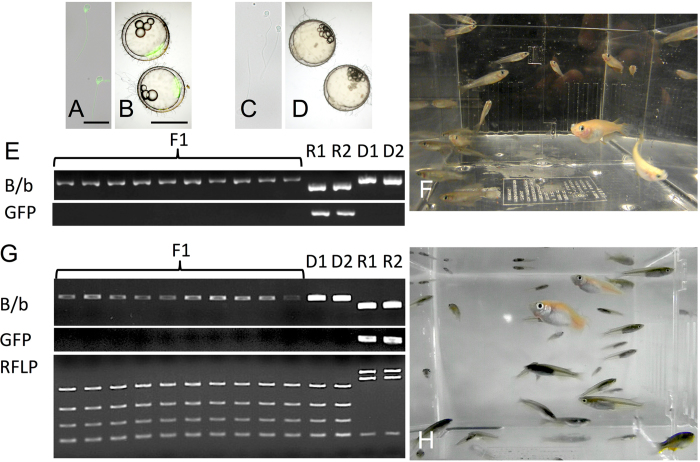
Production of an inbred line (Kaga) and endangered wild population (Tokyo medaka) derived from cryopreserved whole testes. (**A,B**) Sperm and embryos derived from *olvas-GFP* male. (**C,D**) Sperm and embryos derived from *olvas-GFP* triploid recipients after transplantation of cryopreserved Kaga inbred line TCs. (**E**) PCR analysis, performed with *tyrosinase*-specific primers and *gfp*-specific primers, of F1 offspring derived from *olvas-GFP* surrogate triploid recipients by cryopreserved Kaga TC transplantation (lane F1), *olvas-GFP* medaka recipients (lane R1-2) and the donor Kaga inbred line (lane D1-2). (**F**) Medaka individuals (black-colored Kaga inbred line) generated from surrogate triploid *olvas-GFP* parents by cryopreserved TC transplantation. (**G**) PCR analysis, performed with *tyrosinase*-specific primers and *gfp*-specific primers and RFLP analysis for *cytochrome b*, of F1 offspring derived from *olvas-GFP* surrogate triploid recipients by cryopreserved Tokyo medaka TC transplantation (lane F1), donor Tokyo medaka (lane R1-R2), and *olvas-GFP* medaka recipients (lane D1-2). (**H**) Medaka individuals (black-colored wild population of Tokyo medaka) generated from surrogate triploid *olvas-GFP* parents by cryopreserved TC transplantation. Scale bars, 1 mm (**B**), and 10 μm (**A**).

Next, we transplanted cryopreserved TCs cells from an inbred line (Kaga) and an endangered wild population (Tokyo-medaka). Whole testes were vitrified and transplanted into the triploid *olvas-GFP* orange medaka. Although sperm and embryos of the *olvas-GFP* medaka exhibit green florescence (Fig. 4A,B), triploid recipients produced sperm and embryos that did not have green fluorescence (Fig. 4C,D), suggesting sperm and embryos were derived from transplanted TCs. By PCR, F1 juveniles derived from transplanted Kaga-TCs were distinguished with *olvas-GFP* transgenic recipients (Fig. 4E). They produced F1 individuals with black body coloration by mating (Fig. 4F). Moreover, their genetic background of F1 juveniles was confirmed to be identical with that of the donor endangered wild population Tokyo medaka using PCR and PCR-RFLP analysis (Fig. 4G). Thus, we successfully revived an inbred line (Fig. 4F) and an endangered wild population (Fig. 4H) derived from cryopreserved TCs.

### Production of offspring derived from vitrified TCs of a low-fertility strain

**Figure 5:**
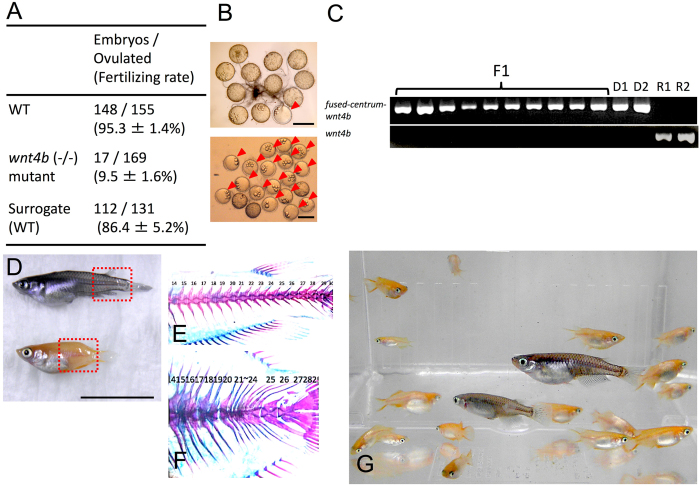
Production of sub-fertile mutants derived from cryopreserved TCs. (**A**) The number of embryos derived from mating with WT black medaka, sub-fertile mutants, and surrogate triploid WT parents receiving cryopreserved *wnt4* mutant TCs in a week. (**B**) Ovulated oocytes and embryos (arrowhead) produced by mating *wnt4* mutants in a day. (**C**) Ovulated oocytes and embryos (arrowheads) derived from mating surrogate WT triploid recipients of cryopreserved *wnt4* mutant TCs in a day. (**C**) PCR analysis, performed with *tyrosinase*-specific primers and normal *wnt4b*-specific primers, of F1 offspring, donor *wnt4b* mutants (lane D1-2), and WT recipients (lane R1-2). (**D–F**) WT medaka (top of D), *wnt4* mutant (bottom of D), microscope sample after skeletal staining for WT (**E**), and that of *wnt4* mutant produced by surrogate triploid WT parents (**F**). (**G**) Medaka individuals (orange colored *wnt4b* mutants) generated from surrogate triploid WT medaka by cryopreserved TC transplantation. Scale bars, 1 cm (**D**) and 1 mm (**B**).

Finally, we propagated the *wnt4b* mutant strain, which has low fertility^25^. In this mutant, the vertebral column is shrunken and the body shape is abnormally short because of mutation of the *wnt4* locus. Because the mutant male cannot hold the female by dorsal and anal fins during mating, their fertilization efficiency is extremely low (5.7 ± 1.5 fertilized embryos/56.3 ± 8.1 ovulated eggs in 4 days, fertilization rate 9.5 ± 2.7%, N = 3, Fig. 5A). Cryopreserved TCs of this mutant were transplanted into black triploid medaka, which have a normal body shape. The black triploid recipients produced numerous embryos with a high fertilization rate (37.3 ± 10.2 fertilized embryos/43.7 ± 9.1 ovulated eggs in 4 days, fertilization rate 86.4 ± 9.0%, N = 3, Fig. 5A), which was not significantly different from that of the WT (which produced 49.3 ± 12.9 fertilized embryos/51.7 ± 13.7 ovulated eggs in 4 days, fertilization rate 95.3 ± 2.5%, N = 3, Fig. 5A). PCR analysis with primers to detect the insertional sequence from the *wnt4* locus showed that all of the F1 offspring produced by triploid recipients possessed the insertional sequence, although the surrogate parents did not (Fig. 5B). The resulting F1 generation exhibited orange body color (231/231, 100%) and their vertebral column was shrunken, which was identical to the phenotype of the donor (Fig. 5C,E). Thus, it was confirmed that the F1 offspring were derived from *wnt4b* mutant donor TCs, and we succeeded in mass production of low-fertility mutants using surrogate parents (Fig. 5F).

## Discussion

This study demonstrated that live medaka individuals could be produced from cryopreserved TCs via intraperitoneal (i.p.) transplantation of their germ cells into sterile triploid hatchlings. The survival rate of recipients after i.p. transplantation of cryopreserved TCs was sufficiently high for practical uses. Further, nearly half of the triploid recipients that survived could produce only gametes derived from cryopreserved TCs. This value is sufficiently high for application in long-term storage of medaka bioresources (Supplementary Table 2). One-year-old Tokyo medaka (an endangered wild population) were included in this study and their offspring were produced from cryopreserved TCs using this technology. Moreover, it is worth noting that the TCs obtained from donor males could produce both functional eggs and sperm. Thus, live medaka individuals were efficiently produced from germ cells retrieved from vitrified testes.

Although triploid males have the potential to produce sperm, the resulting sperm are aneuploid and do not have developmental abilities. In fish, gonadal development is inhibited in the triploid condition. This inhibition is more severe in females than in males. In general, females are completely sterile. In males, on the other hand, although they occasionally produce aneuploid sperm, the embryos produced by these aneuploid sperm could not reach the hatching stage^23^,^26^,^27^. In the present study, although the control triploid males and females did not produce viable offspring, the triploid recipients that received donor-derived TCs produced both *GFP*-positive (donor-derived) eggs and sperm. Further, by mating male and female triploid recipients, the resulting offspring developed and matured normally. More importantly, no F1 offspring exhibited black pigmentation, which was the dominant phenotype in this study. In addition, external morphology was completely normal and their genotype was identical to the donor medaka as well. Therefore, we concluded that the offspring produced by the triploid recipients were completely derived from the donor medaka.

Recipients that received vitrified TCs produced donor-derived gametes had a 74.6% survival rate, and this value was sufficient for practical application of this technology to long-term preservation of medaka bioresources. Although medaka bioresources (inbred line, wild populations, and related species) are bred as live individuals, this system possesses risks, such as accidents at fish-rearing facilities, pathogen infection, and accumulation of mutagenesis. Thus, the germ cell vitrification and transplantation established in the present study are highly valuable for semi-permanently maintaining the valuable medaka bioresources.

In this study, the transplanted medaka germ cells resumed gametogenesis in the recipient’s somatic environment. The testicular cells, presumably spermatogonia, were differentiated into gametes following the sex of surrogates, because oocytes carrying Y chromosome were produced in female recipients. If spermatogonia carrying XY sex chromosomes produced oocytes in the recipient females, then the sex ratio of the F1 generation from the mating of the recipient male and female should be 3:1. To confirm this, we examined the sex ratio of the resulting F1 medaka. The ratio of males to females was 3:1 (male 73.1 ± 0.7% (106/145, N = 3) and female was 26.9 ± 0.7% (39/145, N = 3)), demonstrating that the oocytes produced by the recipient females were derived from spermatogonia carrying XY sex chromosomes. Several previous reports described artificial sex reversal induced by estrogen treatment during sex differentiation before hatching^28^. The expression of DMY/dmrt1bY in the supporting cells is critical for the fate decision of germ cells entering spermatogenesis^29^. Recently, germ cells expressing *foxl3* were reported to differentiate into oocytes and those suppressing *foxl3* expression by male somatic cells differentiated into sperm^30^. This study demonstrated that TCs isolated from sexually differentiated testes contained cell populations that could differentiate into fully functional oocytes by transplantation of TCs into hatching larvae. The results obtained in this study suggested that TCs, probably spermatogonial stem cells, exhibit sexual bipotency and sex of germ cells can be overwritten by the signals from somatic cells.

In some inbred strains (e.g., Hd-rR, HNI, and Kaga, etc.), the entire genome has been sequenced^3^,^4^. However, because their generation time is as short as 1 year, their genome sequences change frequently during the decades of rearing and their fertility is declining because of an inbreeding depression^31^. In this study, the inbred strain Kaga was produced from *olvas-GFP* medaka recipients. Therefore, testes of valuable medaka strains could be vitrified and stored for a long period of time to regenerate live medaka as reference individuals of genome sequencing.

This methodology can be immediately applied on-site for the conservation of endangered wild populations. In fact, although The Tokyo Zoological Park Society attempted to locate wild populations (Tokyo-medaka) in Tokyo, only five populations have been found and other populations have genetic contamination from introduced species. In this study, the Tokyo-medaka was revived from cryopreserved testes using our newly developed germ cell transplantation technology. Because testicular tissue of this local population is currently stored in liquid nitrogen, the Tokyo medaka could be revived anytime, even if the wild populations become extinct.

The method of transplantation of cryopreserved TCs into hatched larvae established in the present study is currently the only available method for long-term, or even semi-permanent, preservation of medaka bioresources. Previously, the cryopreservation of oocytes or early-stage embryos was limited in vertebrates to mammalian species, such as mice and rats^20^. Cryopreservation of oocytes or embryos of fruit fly (*Drosophila*), African frog (*Xenopus laevis*), and zebrafish (*Danio rerio*) are not yet ready for practical use. The main obstacle is that their oocytes and embryos are too large to be cryopreserved. However, germline stem cells, including spermatogonial stem cells, are small enough for cryopreservation of any animal species. Therefore, this surrogate broodstock technology (cryopreservation of germ cells and transplantation into surrogates) is expected to be applicable to other experimental animals.

## Methods

### Fish and Preparation of Testes

Medaka (*Oryzias latipes*) used in the present study were maintained in aquariums under a 14 h light and 10 h dark photoperiod at 26 °C, and were fed 3–4 times a day with finely ground commercial fish diet (Otohime; Nisshin Co. Tokyo, Japan) and once a day with *Artemia nauplii.* Hatching larvae were fed 5 times a day with commercial fish diet (Hikari-labo 130, Meitou-Suien, Aichi, Japan). After anesthetization with 2 ppm 2-phenoxyethanol (Wako, Osaka, Japan) and decapitation, whole testes were obtained from 2- to 4-month-old dominant orange-colored *olvas-GFP* transgenic medaka whose germ cells were labeled with green fluorescence^32^. Wild type (WT) triploid dominant black-colored medaka were used as recipients of germ-cell transplantation. Triploids were induced through heat shock of fertilized eggs at 41 °C for 5 min subsequent to a 2 min post-fertilization incubation at 26 °C using a modification of the protocol established by Naruse^33^. All experiments were approved by the Administrative Panel on Laboratory Animal Care and Use at Tokyo University of Marine Science and Technology. All methods were carried out in accordance with the Guide for the Care and Use of Laboratory Animals from Tokyo University of Marine Science and Technology.

### Cryopreservation of whole testis by vitrification

To optimize cryopreservation protocol of medaka TCs, cryobiological properties were characterized using dissociated cells. Testes of wild-type (WT) medaka were minced and incubated with 0.2% collagenase H (Roche Diagnostics, Mannheim, Germany) and 0.17% dispase II (Sanko Junyaku Co., Ltd., Tokyo, Japan) dissolved in L-15 medium (pH 7.8 with Hepes, Gibco Invitrogen Co., Grand Island NY, USA) containing 10% FBS (Gibco Invitrogen Co.) and 900 U ml^−1^ DNase I (Roche Diagnostics) for 1 h at 23 °C to prepare the testicular cell suspension. The cell suspension was rinsed with L-15 medium containing 10% FBS followed by filtration through a 42-μm pore nylon mesh (NBC Industries) to eliminate non-dissociated cell clumps. The survival rates of medaka TCs were determined by trypan blue staining after each treatment. Non-permeating agents of sucrose induced dehydration of cells and permeating cryoprotectants prevented intracellular ice formation in cells. To determine tolerance of medaka TCs to dehydration, the dissociated cells were exposed to hypertonic solutions (L-15 medium including 0.2–2.0 M sucrose) at 25 °C for 10 min or at 0 °C for 30 min. To determine toxicity of various cryoprotectants, the cells were immersed in each cryoprotectant solution, including 10% (v/v) glycerol (Wako, Osaka, Japan), 8% (v/v) ethylene glycol (Wako, Osaka, Japan), 10% (v/v) propylene glycol (Wako, Osaka, Japan), and 9.5% (v/v) dimethyl sulfoxide (Wako, Osaka, Japan), for 30 min at 25 °C. The permeated cryoprotectants were removed from the cells by rinsing them in L-15 medium including 0.2 M sucrose at 0 °C for 5 min. To determine whether the cryoprotectants listed above could permeate cells, volume changes of cells caused by dehydration in each cryoprotectant solution, including 8% (v/v) ethylene glycol and 10% (v/v) propylene glycol at 0 °C were observed under a microscope (BX51TF, Olympus, Tokyo, Japan) equipped with a BCS 196 cryostage (LK-600PMS, Linkam Scientific Instruments, Waterfield, UK).

Cryopreservation of whole testes was conducted by the vitrification procedure. Vitrification media included 30% (vol/vol) ethylene glycol (EG) or 30% (vol/vol) propylene glycol (PG), 21% (w/v) Ficoll PM-70 (average molecular weight 70 000; GE Healthcare, Uppsala, Sweden) and 0.35 M sucrose (Wako, Osaka, Japan). Whole testes obtained from *olvas-GFP* medaka placed on a copper mesh (Clever, Aichi, Japan) were immersed in 1.5 mL of vitrification medium at 0 °C for 20 min in 2.0-mL Eppendorf tubes (Hamburg, Germany). After removing testes from the medium and cleaning off the medium, they were vitrified by direct immersion in liquid nitrogen. Thawing was conducted by direct immersion with shaking in 0.2 M sucrose solution at 25 °C for 5 s and incubation at 0 °C for 5 min, and testes were collected into L-15 medium including 10% FBS. Fresh and vitrified-thawed testes (Fig. 1G) were minced and dissociated by the method described above. After filtration through a 42-μm pore nylon mesh, the resultant cell suspensions were observed under a fluorescent microscope (BX-51–34FL equipped with U-MWIB2; Olympus). Cryo-injured TCs (presumably membrane damage) resulted in the loss of green fluorescence, whereas living TCs exhibited clear green fluorescence^34^. Furthermore, the majority of cryo-injured cells were lysed by protease activity during the dissociation procedure. Therefore, half of the testes cells was vitrified-thawed and the other was left in L-15 medium including 10% FBS, and cell viability was obtained by the following formula: cell number for GFP (+) in the frozen and thawed half of testes divided by cell number for GFP (+) in the fresh half of testes, multiplied by 100.

### Transplantation of testicular cells prepared from cryopreserved whole testes

To determine the proper developmental stage of recipients used for the transplantation, 3,000 freshly prepared testicular cells were transplanted into the peritoneal cavity of WT hatchings anesthetized with 0.3 ppm 2-phenoxyethanol at 7, 11, 14, 19, or 23 dpf (26 °C). Intraperitoneal transplantation of testicular cells was conducted in accordance with methodology previously described by Takeuchi *et al*.^35^. Further, to optimize the number of donor testicular cells used for the transplantation, 300, 1,000, 3,000, 10,000, or 30,000 of the testicular cells were transplanted into the recipients at 11 dpf. One month after the transplantation, we confirmed whether GFP-positive cells were incorporated into the recipient gonads under a fluorescent microscope (BX-51-34FL, Olympus). The chorion of recipient embryos at 7 dpf before hatching was digested with a hatching enzyme provided by NBRP^36^ to conduct the transplantation.

A total of 15,000 testicular cells taken from cryopreserved whole testes (stored in −196 °C liquid nitrogen tank from 1 h to 1 year) along with freshly prepared control testicular cells were transplanted into the WT triploid hatchings at 11 dpf. To determine incorporation and proliferation efficiencies of GFP-labeled donor TCs in the recipient gonads, recipients were dissected 2 months after transplantation and gonads were observed under a fluorescent microscope (BX-51-34FL, Olympus). In addition, gonadal development of medaka was investigated. Testes or ovaries were fixed in Bouin solution at 4 °C for 2 h, embedded in paraffin wax, and sliced into 5-μm-thick sections. Paraffin sections were immune-stained using mouse anti-GFP antibody (11 814 460 001; Roche, Basel, Switzerland). The primary antibody against GFP was diluted to 1:500, and the secondary goat anti-rabbit IgG conjugated to Alexa 488 (Life Technology, Driverockville, US) was used. The secondary antibody was diluted to 1:200. Further, the same sections were stained with hematoxylin-eosin staining for morphological observations.

### Determination of genetic origin of gametes produced by recipients and progeny tests

Some recipients matured at the age of 2–3 months. Milt produced by recipients was obtained by using abdominal pressure after anesthetization with 0.5 ppm 2-phenoxyethanol. To determine the production of spermatozoa derived from donor vitrified TCs, GFP-fluorescence of sperm was observed under a fluorescent microscope (BX-51-34FL, Olympus). To determine the production of offspring by gametes derived from donor vitrified TCs, triploid recipient males and females were mated. As donor testes were obtained from dominant orange-colored (homozygous; OR/OR) *olvas-GFP* transgenic (homozygous, *olvas-GFP*+/+) medaka^32^ and black-colored wild-types were used as surrogates, all of the F1 larvae were expected to exhibit donor phenotypes (OR/OR and *GFP*+/+) if the recipients produced only donor-derived gametes. Therefore, to confirm whether the F1 larvae were derived from transplanted TCs, body color and GFP fluorescence in gonads of F1 larvae were observed. To confirm genetic origin of F1 juveniles derived from surrogate recipients, total genomic DNA was extracted from their caudal fin and subjected to PCR with each primer. *olvas-GFP* transgenic medaka were used to distinguish donor-derived *olvas-GFP* transgenic medaka and recipient-derived wild-type medaka by the method described by Nakai *et al*.^12^. The same method was also applied to distinguish donor-derived Kaga inbred line or Tokyo medaka and recipient-derived *olvas-GFP* transgenic medaka. For these three combinations, B/b locus was also used to distinguish donor- and recipient-derived offspring using the method of Nakai *et al*.^12^.

Moreover, to confirm the genetic origin for Tokyo medaka, RFLP analysis was performed according to the method of Takehana *et al*.^37^. For *wnt4b* (−/−) mutants, their *fused-centrum-wnt4b* was detected with their specific primers.

### Flow cytometry

To confirm the ploidy of the recipients, erythrocytes collected from the recipients were fixed in 70% (vol/vol) ethanol for 20 min at −20 °C and incubated for a period of 5 min at 25 °C in PBS (pH 7.8) that contained RNase A (10 μg ml^−1^; Sigma) and propidium iodide (200 μg mL^−1^; Sigma). After filtration through a 42-μm pore nylon screen, DNA content was analyzed using a Guava PCA-96 flow cytometry system (Millipore, Darmstadt, Germany).

### Transplantation of TCs from endangered, inbred, and mutant medaka

We investigated the applicability of these techniques to inbred lines, endangered species, and low-fertility mutants. Cryopreserved TCs of endangered species (Tokyo-medaka)^13^ and the Kaga inbred line were transplanted into triploid *olvas-GFP* orange medaka. Cryopreserved TCs of *wnt4b* mutants (fused centrum (fsc) mutant) which have shorter body and low fertility^25^ were also transplanted into WT triploid dominant black-colored medaka.

### Skeletal staining

To identify the *wnt4b*-mutant among the offspring produced by progeny tests using wild type recipients that received the above-mentioned mutant germ cells, skeleton staining was performed. In particular, bony tissues were stained with Alizarin Red, and cartilage was stained with Alcian blue^38^,^39^. Specimens were fixed for 3 days in fixing solution (10% formalin in phosphate-buffered saline) at room temperature. The fixed specimens were washed 3 times in PBS and then their cartilage was stained in 0.1% Alcian blue solution diluted in 70% (v/v) ethanol and 30% (v/v) glacial acetic acid (Sigma) for 30 min. After stepwise washing with 95%, 75%, 50%, and 25% ethanol and distilled water including 30% sodium tetraborate decahydrate, the specimens were immersed in 30% sodium tetraborate decahydrate solution containing 10 mg mL^−1^ trypsin (Worthington, Lakewood, US) at 37 °C overnight to make them transparent. The specimens were washed three times in 0.5% KOH and their skeleton was stained with the solution containing 4% Alizarin Red S (Wako) at 23 °C for 2 h. The stained samples were kept in glycerol and their vertebral column was observed under a binocular microscope (BX-51-34FL, Olympus).

### Statistical Analysis

Data are presented as means ± SEM unless otherwise stated. One-way analysis of variance (ANOVA) followed by the Tukey’s multiple comparison test was used to determine significant differences between group means. Values were considered statistically significant when the calculated P values were less than 0.05.

## Additional Information

**How to cite this article:** Seki, S. *et al*. Production of the medaka derived from vitrified whole testes by germ cell transplantation. *Sci. Rep.* **7**, 43185; doi: 10.1038/srep43185 (2017).

**Publisher's note:** Springer Nature remains neutral with regard to jurisdictional claims in published maps and institutional affiliations.

## Supplementary Material

Supplementary File
