# Supplementary material for: Production of the medaka derived from vitrified whole testes by germ cell transplantation
Source: Sci Rep. 2017 Mar 3;7:43185. doi: 10.1038/srep43185 (PMC5335710; doi:10.1038/srep43185)
Supplement: Supplementary File [file srep43185-s1.pdf]

Title page

The full manuscript title:

Production of the medaka derived from vitrified whole testes by germ cell transplantation

Author: Shinsuke Seki[1,2], Kazunari Kusano[2], Seungki Lee[2], Yoshiko Iwasaki[2], Masaru Yagisawa[2], Mariko Ishida[2], Tadashi Hiratsuka[2], Takao Sasado[3], Kiyoshi Naruse[3], Goro Yoshizaki[2].

[1] Bioscience Education and Research Support Center, Akita University, 1-1-1 Hondo, Akita, Akita 010-8543, Japan

[2] Department of Marine Bioscience, Tokyo University of Marine Science and Tenchology, 4-5-7 Konan, Minato-ku, Tokyo 108-8477, Japan

[3] Laboratory of Bioresources, National Institute for Basic Biology, 38 Saigo-naka, Myodaiji-cho, Okazaki, Aichi 444-8585, Japan

The full author list:

1. (Correspondence author)

First / last name: Shinsuke Seki

Affiliation: Bioscience Education and Research Support Center, Akita University, 1-1-1 Hondo, Akita, Akita 010-8543, Japan.

E-mail address: sseki@gipc.akita-u.ac.jp

Phone number: +81-18-884-6195

2. First / last name: Kazunari Kusano

Affiliation: Department of Marine Bioscience, Tokyo University of Marine Science and Tenchology, 4-5-7 Konan, Minato-ku, Tokyo 108-8477, Japan

E-mail address: reuben.k4649@gmail.com

3. First / last name: Seungki Lee

Affiliation: Department of Marine Bioscience, Tokyo University of Marine Science and Tenchology, 4-5-7 Konan, Minato-ku, Tokyo 108-8477, Japan

E-mail address: skmetany@gmail.com

4. First / last name: Yoshiko Iwasaki

Affiliation: Department of Marine Bioscience, Tokyo University of Marine Science and Tenchology, 4-5-7 Konan, Minato-ku, Tokyo 108-8477, Japan

E-mail address: yoshiko0107@hotmail.com

5. First / last name: Masaru Yagisawa

Affiliation: Department of Marine Bioscience, Tokyo University of Marine Science and Tenchology, 4-5-7 Konan, Minato-ku, Tokyo 108-8477, Japan

E-mail address: masaru.meets.snow@gmail.com

6. First / last name: Mariko Ishida

Affiliation: Department of Marine Bioscience, Tokyo University of Marine Science and Tenchology, 4-5-7 Konan, Minato-ku, Tokyo 108-8477, Japan

E-mail address: blackrose.jyorokua@spice.ocn.ne.jp

7. First / last name: Tadashi Hiratsuka

Affiliation: Department of Marine Bioscience, Tokyo University of Marine Science and Tenchology, 4-5-7 Konan, Minato-ku, Tokyo 108-8477, Japan

E-mail address: tanago_th_1018@yahoo.co.jp

8. First / last name: Takao Sasado

Affiliation: Laboratory of Bioresources, National Institute for Basic Biology, 38 Saigo-naka, Myodaiji-cho, Okazaki, Aichi 444-8585, Japan

E-mail address: tsasado@gmail.com

9. First / last name: Kiyoshi Naruse

Affiliation: Laboratory of Bioresources, National Institute for Basic Biology, 38 Saigo-naka, Myodaiji-cho, Okazaki, Aichi 444-8585, Japan

E-mail address: naruse@nibb.ac.jp

10. First / last name: Goro Yoshizaki

Affiliation: Department of Marine Bioscience, Tokyo University of Marine Science and Tenchology, 4-5-7 Konan, Minato-ku, Tokyo 108-8477, Japan

E-mail address: ymiura0@kaiyodai.ac.jp

Correspondence and requests for materials should be addressed to Shinsuke Seki (email: sseki@gipc.akita-u.ac.jp)

Supplementary Onine Material

Tables S1 and S2

Supplementary Table S1

Efficiency of making triploid recipients and spermatogonia transplantation of donor-derived offspring in each experimental group

Donor *olvas-GFP*, orange		Recipient WT, black	No. of recipient	No. of recipient survived	No. of Triploid recipient	GT of Triploid recipient
**Group**	**Vitrification of testis**	**Heat shock**				
**1**	**No**	**Yes**	**10**	**6/10 (60.0%)**	**5/6 (83.3%)**	**5/5 (100.0%)**
**2**	**No**	**Yes**	**54**	**30/54 (55.6%)**	**1330 (43.3%)**	**8/13 (61.5%)**
**3**	**No**	**Yes**	**41**	**24/41 (58.5%)**	**16/24 (66.7%)**	**13/16 (81.3%)**
**4**	**No**	**Yes**	**36**	**24/36 (66.7%)**	**12/24 (50.0%)**	**9/12 (75.0%)**
**5**	**Yes**	**Yes**	**20**	**13/20 (65.0%)**	**8/13 (61.5%)**	**7/8 (87.5%)**
**6**	**Yes**	**Yes**	**44**	**29/44 (65.9%)**	**16/29 (55.2%)**	**11/16 (68..8%)**
**7**	**Yes**	**Yes**	**47**	**24/47 (51.1%)**	**13/24 (54.2%)**	**10/13 (76.9%)**
**8**	**Yes**	**Yes**	**50**	**29/50 (58.0%)**	**14/29 (48.3%)**	**10/29 (71.4%)**

Supplementary Table S2

Efficiency of making triploid recipients and spermatogonia transplantation and appearance rate of donor-derived offspring among F1 generation

Donor			Recipient			N	No. of recipient survived	No. of Triploid recipient	GT of Triploid recipient	Phenotype of F1	
Group	Body color	Vitrification of testis	Group	Body color	Heat shock					Donor-derived	Recipient-derived
–	–	–	Wild type	Black	No	5	26/97 (36.6 ± 16.0%)	–	–	–	–
–	–	–	Wild type	Black	Yes	8	51/152 (35.7 ± 15.9%)	28/51 (56.8 ± 29.4%)	–	–	–
–	–	–	*olvas-GFP* transgenic	Orange	Yes	5	55/165 (38.5 ± 30.6%)	40/50 (75.4 ± 14.9%)	–	–	–
olvas-GFP transgenic	Orange	No	Wild type	Black	Yes	4	86/166 (49.8 ± 10.6%)	47/86 (58.7 ± 7.3%)	35/47 (63.6 ±17.1%)	–	–
olvas-GFP transgenic	Orange	Yes	Wild type	Black	Yes	4	115/192 (60.9 ± 2.8%)	62/115 (54.8 ± 2.1%)	43/62 (70.0 ± 6.9%)	1169 (100%)	0 (0%)
Inbred line (Kaga)	Black	Yes	*olvas-GFP* transgenic	Orange	Yes	2	46/100 (45.7 ± 3.4%)	35/46 (77.3 ± 6.9%)	20/35 (58.6 ± 23.3%)	50 (100%)	0 (0%)
Endangered wild population (Tokyo medaka)	Black	Yes	*olvas-GFP* transgenic	Orange	Yes	3	28/148 (20.0 ± 6.2%)	9/20 (37.8 ± 21.1%)	6/9 (63.9 ± 12.7%)	50 (100%)	0 (0%)
Line has low fertility (*wnt4b* -/-)	Orange	Yes	Wild type	Black	Yes	3	41/155 (29.2 ± 1.7%)	25/41 (61.4 ± 3.4%)	21/25 (83.3 ± 11.0%)	259 (100%)	0 (0%)
